# Lost in Translation: State Policies and Micro-politics of Water Governance in Namibia

**DOI:** 10.1007/s10745-016-9820-2

**Published:** 2016-04-09

**Authors:** Michael Schnegg

**Affiliations:** Universität Hamburg, Hamburg, Germany

**Keywords:** Natural resource management, Water, Institutions, Governance, Namibia

## Abstract

Water governance in rural Namibia has profoundly changed since the early 1990s. After independence and in accordance with global environmental policies, it became a central theme of Namibia’s environmental legislation to transfer the responsibility for managing natural resources to local user associations. In this article, I explore the emergence of new social forms at the intersection of existing cultural models and new rationalities for governance. Doing so combines an analysis of state legislation with the micro-politics of water governance in 60 pastoral communities. The ethnographic analysis reveals that different actors, including state bureaucrats as well as rich and poorer herd owners, have different understandings of how to share water. While the poorer often agree with the state policy that water is an economic good and should be paid for accordingly, only in about half of the communities do corresponding institutional regimes emerge. Using critical institutionalism as a theoretical guide, I offer a contribution to understanding how more than 20 years after Rio local institutions of resource governance emerge at the intersection of different, and often heterogeneous and intertwined, social fields.

## Introduction

In the arid environment of northwestern Namibia, almost all households own cattle and small stock. Water and land are the two central natural resources, and until the 1990s, their access was regulated by the South West Africa administration under the jurisdiction of the colonial South African state. Under this administration, local level water governance was largely in the hands of political authorities, including big men,^1^ headmen, and chiefs. Since the colonial state covered the costs of running and maintaining the infrastructure, little local coordination was required. Water was basically free.

Starting in the early 1990s, water governance in rural Namibia has profoundly changed. After independence and in accordance with global environmental policies, it became a central theme of Namibia’s environmental legislation to transfer responsibility, costs, and benefits of resource management to local user associations. The process goes hand in hand with a redefinition of the role of the state. While the state pulled back from governing natural resources actively, governing authorities initiated far-reaching legislation that circumscribes communities’ ways of doing so. The legislation is founded on concepts like Community-Based Natural Resource Management (CBNRM) and Integrated Water Resources Management (IWRM) that contain clear premises about the nature of resources and their appropriate uses (Barnes *et al.*^2002^; Hoole 2009; Jones and Murphree 2001; Schnegg and Bollig 2016; Silva and Mosimane 2013; Vette *et al.*^2012^). The implementation of CBNRM and IWRM led to a drastic reconfiguration of the institutional landscape in rural Namibia and hundreds of communities had to find “new” ways for governing one of their two salient natural resources, water (Bollig and Menestrey Schwieger 2014; Falk *et al.*^2009^; Menestrey Schwieger 2015; Schnegg and Bollig 2016; Schnegg *et al.*^2016^; Schnegg and Linke 2015).^2^

The aims of this article are to explore (1) which institutions of water governance emerged at the intersection of new rationalities for governance and existing social and cultural forms following independence in 1990 and to explain (2) under which circumstances one or the other institutional regime is more likely to develop and prevail. To answer both questions, I introduce a theoretical framework that addresses the negotiation of institutions at the intersection of state policies and communal micro-politics. Then, I present the research design, the data, and the study sites. The remaining sections show which institutional blueprints are carried into the communities and how and why these are implemented or not.

## Theoretical Framework

I use a number of related theoretical concepts as a guide to explain the emergence of “new” social forms at the intersection of different rationalities for governance. Moore has defined a *semi-autonomous field* as a social setting including different actors who can generate rules and coerce compliance with them to analyze comparable situations (Moore 2000:57). Social fields are semi-autonomous because they are embedded within larger legal and political frameworks and connected with one another. In Moore’s analysis, the state plays a major role. Moore argues that legislation is the most usual way in which central governments invade social fields within their political boundaries. Although laws are often passed to alter existing social arrangements, traditional “social arrangements are often effectively stronger than the new rules” (Moore 2000:58).

Building on Moore’s work, Lund has explained that public authority is not an either or of different rules but often a hybrid of state policies and local practices (Lund 2007a, 2007b, 2011). He coined the term *twilight institutions* to refer to processes that cannot be understood by focusing on “the state” or the state-society dichotomy alone (Lund 2007a:15). Rather, and as Abrams has shown so convincingly, the state is both an idea and a system (Abrams 1988). These elements of the state are not given but made in daily routines and by a wide range of actors. At the intersection between state and society, public and private, and negotiated by a variety of actors *twilight institutions* emerge often containing and translating ideological elements of the state to other public domains (Lund 2007a).

Extending the analysis beyond the relationship between society and the state, Cleaver has argued that institutions are embedded in a range of social and cultural fields (Cleaver 2002, 2012; Cleaver and de Koning 2015). For her, embeddedness has two dimensions: First, Cleaver argues, rules are transferred from one social field to another, e.g., people build new institutions based on their experience with existing ones. Second, rules do not fulfil one purpose alone, i.e., rules formulated for managing water do not just manage access to and distribution of water but may equally control land and membership in social groups (Cleaver 2012).

Moore, Lund, Cleaver, and other scholars (De Koning and Cleaver 2012; Hall *et al.*^2014^; Leach *et al.*^1999^; Mosse 1997; Saunders 2010, 2014) share a set of central assumptions that guide my analysis: First, a social field like water management is not fully autonomous. It is embedded in different normative orders and locally situated forms of sociability. Those normative orders may be structured by state sponsored legislation with the goal of regulating the ways communities govern their resources (e.g., water). Alternatively, existing social institutions may provide procedures intended to regulate different or interconnected social fields (e.g., sharing food or land) (Schnegg and Linke 2015) and these may be extended to or adapted for water management. Secondly, heterogeneous social actors, including resource users, state bureaucrats, and NGO representatives, negotiate both sets of processes.

While these approaches put the embeddedness of institutions center stage, they do not explain when and why one set of rules becomes salient or not. As I show here, theoretical modes developed at the intersection of social anthropology and new institutional economics can account for these variations (Acheson *et al.*^1998^; Ensminger 1992, 1998; Haller *et al.*^2013^; Menestrey Schwieger 2015). For new institutional economics, transaction costs and bargaining power are central concepts (Ensminger 1998; Knight 1992). *Transaction costs* refer to the costs involved in carrying out an exchange, e.g., the costs involved in getting information about the availability and quality of a good, transportation, etc. Institutions reduce transaction costs, when they make behavior predictable. Theoretically, actors favor institutions that lower (their) transaction costs and institutions develop in a direction that lowers overall costs involved in making exchanges. Furthermore, each individual opts for institutional solutions that serve his or her distributional goals (Ensminger and Knight 1997; Knight 1992; Knight and Sened 1995). The more *bargaining power* an actor has, the more likely they can establish his or her preferred rules. To summarize, the goals and interests of an actor in combination with their bargaining power and the costs that an institutional regime is likely to produce can help to explain why one set of rules is established and another not.

## Methods

### Research Design and Data

The data analyzed here were collected in northwestern Namibia by a team of anthropologists between 2010 and 2016 (M. Bollig, M. Schnegg, Th. Kelbert, D. Menestrey, Th. Linke, K. Gradt) as part of a German Research Council (DFG) funded research project *Local Institutions in Globalized Societies* (LINGS) (Bollig and Menestrey Schwieger 2014; Schnegg 2015; Schnegg and Bollig 2016; Schnegg *et al.*^2016^; Schnegg and Linke 2015). The two principle investigators, Schnegg and Bollig, have been conducting ethnographic fieldwork in the region since 1994 (Bollig) and 2003 (Schnegg), respectively, and are responsible for the overall design and comparative analysis of the data. In the first phase of the current fieldwork, three anthropologists (D. Menestrey, Th. Linke, K. Gradt) stayed for roughly one year between 2010 and 2011 in the southern (Fransfontein), central (Otwani) and northern (Okangwati) parts of the research area to get an in-depth understanding of processes entailed in negotiating and crafting new institutions through daily routines. The qualitative data presented below were collected as part of ethnographic fieldwork and stem from all three research sites.

After an initial analysis of the ethnographic data collected during 2010 and 2011, we returned to the field in late 2012 to conduct the up-scaling research we had designed to study the distribution of some of the phenomena found in the community ethnographies. Since our study treats communities as cases, it is costly and methodologically challenging to get relatively large numbers of observations that permit meaningful comparisons. To achieve this end, we designed an interview guide to elicit information at the community level. For geographical areas of approximately 250 km^2^ surrounding Fransfontein, Otwani, and Okangwati, 60 communities were sampled concentrically around the localities we had researched in depth in preceding years. We decided against a representative sample of the entire Kunene Region due to the size, bad road infrastructure, logistical constraints, and a lack of a list of communities that could serve as a sampling frame. In addition, our approach allowed making use of the fact that fieldworkers were already known and trusted in the target areas.

During the community visits, we elicited rules of water management and the composition of community-based organizational structures for water governance. Each focus group discussion took place in public and included both female and male informants of whom some were active in the committee responsible for community-based water resource management. The focus group discussions were semi-public so that people could join in during a session. In most cases, it took two days to complete a focus group discussion in any one community. In total, we visited 60 communities. Since information remains incomplete for some of them, the quantitative analysis presented here considers 56 cases.

A significant part of the data was already coded when entered into the database designed for our project purposes. The data set incorporates more than 50 variables that contain, for example, information about institutional regimes, institutional history, technological infrastructure, population size, demographic composition, and livestock ownerships. Although most of the variables were already coded in the survey, two variables that we consider in this analysis were not: the rules for water management and the involvement of the state. For the coding, I categorized the rules for contributions from community residents into those proportional to use and those where this is not the case (see below). The coding was cross-checked by the other members of the research team. To measure the involvement of the state, we use two indicators based on information collected in the focus groups. First, we asked about the frequency and purpose of visits by state officials to the community during the last two years. When state officials visited a community at least once during the previous year for consultancies and activities other than urgent repairs of broken infrastructure, this served as an indication of more than average state involvement. Generally, we found state officials want to push communities in certain directions, above all concerning the payment schemes for water use. Second, our ethnographic observations showed that some employees of the ministry or commissioned NGOs lived in the communities at issue. In these cases, the impact of the state was significantly stronger because those people typically wanted their communities to be flagship cases for the state’s mission and ideology (see below) and thus worked consistently with other residents in the hope of achieving this. For the analysis, we coded state involvement to be above average, if either of the two indicators (more than one visit per year or a state authority in residence) was given.

### Study Sites

Pastoralism is the dominant subsistence strategy throughout the research area, and dependency on natural resources is high. Like water, pastures are communal property. But in contrast to water, the organization of grazing does not incur monetary contributions and the cost-sharing arrangements that we describe for water do not apply here. With grazing, land boundaries and access are salient and typically regulated by local communities under the auspices of traditional authorities, land boards, and chiefs.

In arid Kunene, pastoral livelihood is constrained by the environment, most notably the low and unpredictable precipitation (Bollig 2006; Schnegg and Bollig 2016; Schnegg *et al.*^2013^). Annual rainfall varies between 200 and 300 mm and occurs in summer, between November and April. In the arid environment, access to more than 25–30 ha of land is needed during a year to sustain one head of cattle (Burke 2004). A head of cattle drinks about 27 l and goats/sheep 2.2 l (Wilson 2007:60f) of water each day. For comparison, humans use about 20 l of water for drinking and sanitation per day if water has to be carried to the house.^3^ With large cattle herds, the amount of water used for animals is thus significantly higher than that which humans consume.

While pastoralism is the dominant subsistence strategy in the area, the region is heterogeneous in terms of ethnicity and the ways pastoralism is practiced and integrated into the labor market. Even though an ethnographic sketch here must remain superficial, I offer a glimpse of the local context as background. Various ethnic groups including the Damara, Nama, Ovaherero, and Ovambo inhabit the communal settlements in southern Kunene, located near Fransfontein. Under South African rule, the area was part of the so-called Damaraland homeland. In contrast to the other research regions included, Fransfontein has a more favorable connection with Namibia’s urban centers, and wage labor plays a significant role in household economy. Households usually possess less livestock than further north, and people make use of diverse economic strategies to make a living. Nonetheless, pastoralism still shapes daily life in the communal settlements (Greiner 2011; Pauli 2011; Schnegg *et al.*^2013^). The area of Otwani, situated halfway between Sesfontein and Opuwo, is mainly populated by the Ovaherero people. During South African colonial rule, this region was part of the so-called Kaokoland homeland. People there mainly make a living through pastoralism and seek access to local livestock sales markets, although there is also a partial integration of this area into the wage labor market. In the northern most region around Okangwati, pastoralism again is the dominant economic activity. Although also part of the former so-called Kaokoland, the colonial rule was less direct here than in Fransfontein (Bollig and Menestrey Schwieger 2014; Menestrey Schwieger 2015). Even though the research area is ethnically diverse and pastoralism is practiced with different levels of market integration, the practices of providing water and grazing are comparable throughout the area. Similar ecological constraints and organization around drilled boreholes largely shape the ways water is managed.

People use three strategies to acquire water: (1) constructing dams along seasonal rivers, (2) digging holes into the sandy beds of the rivers where the water stays long after the river has stopped flowing at the surface, and (3) drilling boreholes to access the groundwater. The technological infrastructure for these boreholes is heterogeneous: windmills, hand pumps, electronic motors powered by solar panels, and—by far the most important—diesel engines.

All water sources but boreholes support only a relatively low population and livestock density. To populate Namibia’s rural hinterlands and to compensate for demographic growth, the state increased the number of boreholes in the northern region of Kunene by a factor of almost ten between 1960 and 1990 (Bollig 2013:323). Like in other parts of Africa, this hydrological perforation of the landscape has changed usage patterns and fostered a more sedentary life style (Gomes 2006; Picardi and Seifert 1977). Today, water is almost exclusively provided through boreholes during the dry season. All members of a community have access to the water that is pumped into large, open reservoirs. During the rainy season, dams and dug holes supplement but do not replace the borehole water supply. In a nutshell, rural communities in Kunene are highly dependent on boreholes and their management.

## Results

### State Policy

Following the Rio 1992 summit (The Earth Summit), resources governance in the global South profoundly changed. Decentralization and community-based resource governance became a salient part of the international development agenda (Falk *et al.*^2009^; Furlong 2010; Taylor 2007). Most importantly, “The Earth Summit” introduced 27 principles that promote sustainability, poverty eradication, and participation (Little 1995; UNDSD 1992). For water, Agenda 21 formulates that its governance should reflect “as far as possible both the true cost of water when used as an economic good and the ability of the communities to pay” (Agenda 21: Chapter 18). Equally, the *International Conference on Water and the Environment* (ICWE) that met in Dublin the same year proclaims in one of its four principles that “water has an economic value in all its competing uses and should be recognized as an economic good.”

In 1993, the *Water Supply and Sanitation Sector Policy* (WASP) of Namibia translated these ideas about water management into the national context defining “community ownership and management of facilities” as a guiding principle of water management for the rural sector (Namibia 1993). Soon after, the *Water Supply Coordination Committee* (WASCO), an organization established to coordinate the process and to recommend principles for the Namibian situation, formulated that “water is an economic good and that consumers shall contribute for water supply services” (Namibia 1997). In practice, the new policy for the rural water sector led to a significant and enduring effort of the state to pass the responsibility of boreholes and their infrastructure to local user associations (Falk *et al.*^2009^; Gildenhuys 2010), a process envisioned to lead to the “empowerment of the people and the transfer of authority and assets to the community” (Namibia 2006:16).

The National Plan includes three steps: firstly, a phase of sensitizing and mobilizing the communities in CBNRM related matters and an update of the technical infrastructure (phase 1: capacity building); secondly, the rehabilitation of water points selected for the implementation of CBNRM, the establishment of Water Point Associations (WPA) and Water Point Committees (WPC), and the “training” of local communities to develop and formalize rules of water governance (phase 2: operation and maintenance); and thirdly, shifting the ownership of government assets to the WPA. The entire responsibility for all aspects of managing the water infrastructure is turned over to the community including the replacement of major technical equipment (phase 3: full cost recovery). To standardize the process, a *Handbook for Water Point Committees* (Namibia 2006) was developed and distributed by the state to the NGO and state representatives administering the process on the local level. The handbook and related documents describe the process to be taken step by step in 11 chapters and propose institutional solutions to the community.

Sharing the costs of water is one of the most salient problems in water governance. Since most pumps operate with diesel, the price of water is largely determined by the amount and price of diesel required for pumping it. In chapter 5, “Managing WPA Finance,” the handbook reads: “Recommended is a rate per head of large or small stock, each member paying a certain rate per head of large or small [stock] accordingly, as to raise enough money to sustain the water point” (Namibia 2006:8). We refer to this arrangement as the “per-head-of-cattle rule” (p.h.o.c.-rule). In addition, a second payment scheme is introduced. It recommends equal contributions for all households or individuals, mostly to cover the costs of domestic usage. Following the wording of the handbook, we call this the “flat rate rule.” In practice, the institutional solutions to cover the costs of water oscillate between these two extremes. In the former case, contributions are regulated according to the amount of water used. In the second case, payment for water is a fixed sum and more independent of usage.

During the process of implementation, emerging institutional arrangements are negotiated with representatives of the ministry or contracted NGOs. Representatives visit the communities and call for meetings during which many pertinent questions, e.g., access, sanctions for violations of the rules agreed upon, and—often most importantly—payment schemes are discussed. Since discussions about the payment schemes are typically conflict-ridden, the process often requires a number of meetings that stretch over months (Schnegg and Bollig 2016; Schnegg *et al.*^2016^; Schnegg and Linke 2015). During the meetings, state representatives take an active role. They go through the sections of the handbook and sensitize the communities to the issues they have to resolve. While they do not provide any material incentives to apply a p.h.o.c.-rule or penalize communities that opt for flat rate rule, the state’s representatives clearly articulate in public meetings that the p.h.o.c.-rule is what the state favors and what they perceive to be just and fair.

### Micro-politics of Water Governance

As we have seen, the government promoted a proportional p.h.o.c.-rule through its bureaucrats. At the end of the process, in which those rules were negotiated, all communities had to agree upon a management plan, officially signed by the members of the committee. The management plan gives a tentative budget for a water point and specifies rules for how each member should contribute financially. Equally, the plan defines sanctions for those who do not comply. In the vast majority of the management plans, communities formally subscribed to a p.h.o.c.-rule that all households should pay according to the livestock owned.

However, as interviews and observations reveal across the three research sites, in many communities, the rule was never applied or, if it had been, it was altered relatively soon thereafter. When we conducted the up-scaling research in 2012, only 25 (44.6 %) out of the 56 communities had continued with a p.h.o.c.-rule. In addition, seven communities (12.5 %) use an attenuated form in which the rich pay more but not exactly according to their number of livestock (e.g., rich = 200 N$, poor = 100 N$). This difference in payments is not as significant as the differences that emerge with exact livestock counts. In 24 communities (42.9 %), the proportional rule, promoted by the state and NGOs, was never practiced or replaced by an institution that requires equal payment for all households (Schnegg *et al.*^2016^). In some of these cases, money was collected and a trusted person bought diesel with it. In equally many cases, the households had agreed that each household would pump for a month or that the households in charge in that specific month would provide the diesel to a caretaker in charge of pumping the water. All solutions lead to equal pay (flat rate rule).

Before we offer an explanation for the institutional diversity observed, we want to rule out two alternative hypotheses: technology and a “majority vote” (Anderies *et al.*^2004^; Ostrom 1990). Whereas Anderies *et al.* (2004) have proposed that technology plays a central role in institutional design, we find a low and non-significant relationship between the technological infrastructure (solar/diesel) and the payment regime (phi=0.155, sig=0.244). Alternatively, Ostrom has argued that institutions tend to develop in ways that serve the majority (Ostrom 1990:193). In the Namibian case for water management that would mean that households would opt for the solution under which they would pay less (given their number of animals) and that a solution would prevail that serves most households best. A test of the second hypothesis reveals that in most communities (88.9 %), the majority of households would profit from a p.h.o.c.-rule. Thus, the hypothesis fails to explain why in a great many communities the flat rate is carried out.

The account we provide instead takes into account that (1) a p.h.o.c.-rule produces higher transaction costs than a flat rate rule, (2) the rich and powerful favor the flat rate rule and profit from it, (3) water management overlaps with other social fields, and (4) the state and its bureaucrats play an active role.

### Transaction Costs

To establish and maintain a payment regime requires (1) collecting and storing money, (2) paying for diesel and repairs, and, with a p.h.o.c.-rule, (3) counting animals. These costs involved in implementing a rule are part of the transaction costs. There are social costs associated with collecting and keeping money. Social capital and trust are required. Typically, the person assigned as treasurer keeps the money in the household and hands smaller sums to the technician/pumper to hike to the next petrol station to buy diesel. In 65 % of the cases (*N* = 43), we were able to document that a woman fulfils the role of treasurer for the water point committee. Asked why, people readily explain that women, who typically manage household budgets, are more trustworthy, experienced, and also less mobile than men. These traits reduce the probability that the money will be spent for other purposes. These relatively high social costs are one reason some communities apply a flat rate rule without collecting money and take turns among the households pumping water.

There are also costs associated with counting animals. Counting 100 or even more animals is extremely laborious. Typically, livestock from diverse herd owners come to drink at a water point the same time and it is often challenging to direct, control, and thus count them separately. Beyond that, many Ovahimba and some Ovaherero people explained to us that animals will die once enumerated. This taboo puts an additional burden on practicing animal counts.

To summarize, the costs for collecting and keeping money and for buying diesel do not differ between flat rate and p.h.o.c.-rules. But, there are differences in the ways a flat rate rule can be implemented to ameliorate social costs: cash based (assuming those costs) or labor based (avoiding the transaction costs). In contrast, the p.h.o.c.-rule requires counting animals, which comes with burdensome labor and the costs of working against a cultural taboo. Both institutional regimes provide for an option in which transaction costs rise above simply pumping in turns, i.e., collecting the used money based on flat rate or proportional payment regimes. The more costly a regime, the more difficult it is to establish and maintain.

### Wealth, Bargaining Power, and Institutional Preferences

Economic capital is unevenly distributed in the Kunene region (Pauli 2011). While we find in almost all communities at least one household that owns more than 100 cattle, we also find at least one, and often more, that owns less than 10. These economic differences largely shape the social structure of the communities and often result in hierarchical social relationships. In ethnographic interviews and through observing public discussions about the most appropriate institutional regime for water management, we found evidence for a correlation between wealth in cattle and preference for a particular rule. Across all three research sites, wealthy herd owners oppose a p.h.o.c.-rule and opt for a flat rate solution (Menestrey Schwieger 2015; Schnegg *et al.*^2016^). In this context, we use the terms *wealthy* and *poor* as relative categories to distinguish the upper quartile of the economic spectrum from the rest. Often, those are only one or two families who own more than 100 head of cattle. The following excerpt from an interview with Hermann, an elderly and wealthy cattle owner from one of the communities surrounding Fransfontein, provides an example of the perspective of the wealthy and offers some insights into the logic of the argument:

Let me put it this way: Jorries owns fifty cows and I own five. Then the people will say: “Why is Jorries only giving 20 Namibian Dollars, and I am also giving 20. The person with more cattle must give more money.” (…). [However,] the water point belongs to all of us. We must understand each other. Today you have five cows, tomorrow they can become ten, and then Jorries, due to the theft plaguing us, may soon loose all his animals. There is one more thing that needs to be considered. Jorries who is having fifty cows is not only keeping them for the water, but he is also taking milk from his animals and gives this to you so you can prepare some porridge and eat it with the milk.^4^

Hermann, the wealthy pastoralist cited above, not only offers a clear rejection of a p.h.o.c.-rule but also provides a justification. First, he refers to the fluctuation in herd sizes. According to his logic, the rich may be poor tomorrow and vice versa. Why should community members then care about complex rules, counting, and proportional thinking? Hermann goes on to argue that those who own more cattle also have more milk to share. As I explain in more detail below, the exchange of milk is salient to the livelihoods of the young and the poor and establishes both an economic and a social relationship. Hermann thus embeds the sharing of water into others transactions and argues that they will balance out across different exchange spheres and time.

However, the majority of people have a different view. In contrast to the wealthy few, they plead for a p.h.o.c.-rule. The following section from an interview with Tobias, a relatively poor and younger head of household from the northern-most research site, makes this clear. He argues:

To pay according to the amount of cattle is very good. It is fair because everyone gets his own water. If I have five head of cattle and you have a hundred, but both pay the same, the rich one is free because he is using my money. And I have to pay more than I have—that means that is not fair.^5^

In the interview, Tobias argues elaborately against an equal pay plan and points out that those who own more must also pay more.

In the research area, age, status, and bargaining power are typically correlated. However, while most wealthy people are older, not all elders are rich. The preference for the institutional regime is strongly linked to the livestock ownership and we did not meet any member of the economic elite who opted for a p.h.o.c. rule. In Kunene, the stark economic stratification often results in hierarchical exchange relationships. For example, elder Ovahimba and the Ovaherero tend to lend livestock to younger men, often matrilineal relatives, who in return receive the right to utilize the milk. On average, more than 10 % of the cattle are leant to other households and young herders even borrow 95 % of the animals they stay with (Bollig 2000a). In contrast, wealthy and elder households own almost all of livestock in their kraal (an enclosure for cattle or other livestock).

In addition to livestock ownership, the Namibian state provides all citizens aged 60 and above with a pension providing enough to buy the basic necessities. In a poverty ridden environment, even this small amount of income can establish an economic position of superiority (Schnegg *et al.*^2013^). To summarize, wealthier people prefer the flat rate rule. Typically, they are also community elders. As we will see, those who own more use their social status to push an institutional regime that is favorable for them (flat rate). The nature of social ties is key to understanding why the wealthier often succeed.

### Social and Cultural Embeddedness

The communities in Kunene are relatively small and social embeddedness is high (Bollig 2000b; Schnegg 2015; accepted; Schnegg and Linke 2015). Dense and multiplex social networks constitute a specific social fabric with far-reaching consequences for building and maintaining institutions, as the following case of Justus and his uncle reveals.

On Vingerklip,^6^ a community in the Fransfontein area, Alfons, the head of the wealthiest household, refused to pay more than the rest and thus to support a p.h.o.c.-rule. His nephew Justus is the chairperson of the water point committee. When our talk came to water, Justus bitterly complained about his uncle and his refusal to pay according to his number of cattle owned. When I asked him, why he, as the elected chairperson, and the majority of the households could not push through their understanding of a just and fair rule, he explained:

Since we were young, we knew exactly, who is who, and that you have to have this respect for, let us say “this is my uncle, I can’t talk to my uncle like that.” He is the big one in the family who’s running the house, is the one making the decisions. I must not talk against my uncle. So, if my uncle says something, whether it is right or whether it is wrong, I have to follow the rules. So, that causes a lot of problems in the community, because now, the families were afraid to talk to their uncle, because they were keeping this respect.^7^

Justus explains that the dense kinship network and the normative expectations that come along make it impossible to force his uncle to accept a particular position. Among all kinship ties, that between a nephew and his uncle (mother’s brother) is one of the most salient. A man will inherit property (especially cattle) from his maternal uncle and is supposed to be subordinate, helpful, and respectful to him. In Kunene, kinship, generations, and gender structure social interactions to a large degree and as the case study reveals, water management is part of the wider social field.

In recent years, Cleaver has argued convincingly that resource governance is not only socially but also culturally embedded (Cleaver 2012). She coined the term *institutional bricolage* to indicate how “new” rules built on existing forms. With the case analyzed here, this becomes evident when we look at the justifications people bring forward for focusing on the central cultural unit, the household (Otjiherero: *oanda*, Khoekhoegowab: *||gaus*), instead of counting animals.

All we normally do is to contribute per household. Therefore for us it is never a problem, because we are used to the issue of receiving something per household or paying, contributing per household. Even as we are talking now, if I slaughter cattle, and I divide this meat among the households I will divide it per household without considering how many people there are. Many of the things or activities we do it per household. That is our normal routine, that’s why it was never a problem for us, since we are used to this practice of per household.^8^

Here, Pete, a relatively rich household head, argues that for sharing water rules should apply that work in other cultural fields as well. When benefits like meat are shared, this is not done in proportion to needs but instead all households get the same. The same logic, he continues, should apply for cost sharing as well. Both ethnographic vignettes show that institutions are not isolated form the wider social fabric and that the rules that emerge can only be understood if we take this social and cultural embedding into account.

Given the three dynamics just described, (1) higher transaction costs, (2) individual preferences, and (3) the social embeddedness of water management regimes, we would expect all communities to shift from the p.h.o.c. to a flat rate rule. All three dynamics work in the same direction. However, and as we have seen, this is not the case.

### Linking State Policies and Micro-politics

To answer why, I ask when the institutions emerge that the majority claims to be just and fair. A closer examination of those cases reveals that they share a key characteristic. State ministries and NGOs contracted by the state maintain strong involvement in the local water governance in these cases. As we have seen before, not only local actors have different interests and bargain about the proper institutional solution but, in these cases especially, the state and its local representatives do so as well (Menestrey Schwieger 2015; Schnegg *et al.*^2016^).

An interview with Martha who works for the Ministry of Agriculture, Water and Forestry and is responsible for roughly 100 water points shows the state at work. When we talked about my observation that most communities switch to a flat rate rule, she responded: “It is not fair. But as soon as we turn our back the community big men come and tell the rest what to do.” In the course of the interview, she repeatedly states how frustrated she is that she, the official from the Ministry, cannot even implement the rule in the community where she is farming herself. Asked where the p.h.o.c.-rule is actively working, she starts talking about the community Duurwater where an active young woman is the chairperson. To support her, Martha drove early in the morning, when the cattle drink, to Duurwater to count the animals with the other committee members. “Then, we approached the poor households and talked to them about the different rules and encouraged them to stand up and talk in the meeting. In the meeting, we would support them.”^9^

Much of the institutional reconfiguration in rural Namibia takes place in a social environment where the state has explicit preferences and at least some means to pursue them. The institutional regimes that emerge can only be understood if we consider the distribution of power at the local level and the role of the state simultaneously. Recognizing this allows us to formulate a hypothesis, which combines the arguments and observations made so far: Communities will employ a flat rate rule unless the state actively supports the less wealthy pastoralists and their interests.

Table 1 tests this hypothesis and shows the correlation between the institutional regime (flat rate versus p.h.o.c.-rule) and the involvement of the state coded as discussed above. The results clearly show that the involvement of the state can explain to a significant degree the institutional outcome (phi = −0.478, sig = 0.000).^10^ This confirms that in communities where the state is only weakly involved a flat rate rule is more likely to emerge. In those communities, the three dynamics analyzed above push the institutional regime toward a flat rate rule.

**Table 1:** Engagement of the state and institutional regimes (*N* = 56, phi = −0.478, sig = 0.000)

	Water institution		
State/NGO involvement	Flat rate	p.h.o.c.-rule	Total
Strong	4	20	24
Weak	20	12	32
Total	24	32	56

An institutional form that diverts from the main pattern offers additional insights into the dynamics observed. In seven communities, an attenuated p.h.o.c. rule is practiced and the rich pay twice as much as the poor. A closer examination of these communities reveals that the state is comparably inactive (coded as “weak”) in six of them (85.7 %). They thus lack the external support to establish a p.h.o.c. rule. In Table 1, all six cases fall into the category of proportional rule and weak state involvement and make up half of the 12 communities here. This rule goes part way to meet governmental and common fairness concerns while avoiding the transaction costs of animal counts. This *twilight institution* (Lund) that blends two forms appears to be a compromise that local power dynamics allow for.

## Discussion

Currently, water governance in rural Namibia is changing profoundly. The process is in accordance with many post-Rio environmental policies and was initiated by the Namibian state that withdrew from financing water directly while formulating clear specifications on how local institutions should develop (Falk *et al.*^2009^). After intense discussions, most people in the research area agree with the state’s preference that the best choice is to pay according to livestock ownership (p.h.o.c.-rule). However, in many communities, this rule does not emerge or prevail and “social arrangements are often effectively stronger than the new rules” (Moore 2000:58). To understand why, we have to take into account (1) the transaction costs involved in establishing a rule, (2) the particular interests of rich and poor pastoralists, (3) the embeddedness of resource management, and (4) the role of the state.

The analysis shows that transaction costs gradually rise from pumping water in turns, to collecting money for flat rate contributions, to an attenuated p.h.o.c. rule, and a proportional payment regime that involves animal counts. The higher the costs, the more difficult it is to establish and maintain the institutional regime. As we have seen, people seek an institutional regime that serves their material ends; while the poor favor a proportional payment, the rich opt for flat rate rule (Knight 1992, 1998; Knight and Sened 1995) and what ultimately emerges reflects power dynamics among community residents and the role of government or NGO representatives.

Cleaver has explained that rural water management is socially and culturally embedded (Cleaver 2001, 2012; Cleaver and Franks 2005; Cleaver and de Koning 2015). Following what Cleaver coined an *institutional bricolage*, the rich use sharing models that apply in other cultural domains to justify their cause. In Kunene, sharing water cannot be separated from sharing ancestries, food, and grazing, and salient kinship relations overrule and restrict behavior in other social domains. Even if the poor are in the majority, social embeddedness works in support of the elite and makes it hard or even impossible to influence or even sanction them (Schnegg and Linke 2015). Left alone, the rich typically succeed in establishing a rule that favors them.

This finding is supported by a number of studies about the decentralization of forestry. For example, Kumar has shown the consequences new forms of participation can have. Among forest users in India, he finds that “new” regimes reflect the social preference of the rural non-poor and that the poor are likely to lose in the long run. As in the case presented here, existing power structures and inequalities are determining and readily reproduced (Kumar 2002). In a similar vein, Kumar, Singh, and Kerr (2015) have reported that the implementation of democratic decentralization of natural resources governance has been strongly opposed by powerful interests and that many of its provisions related to community rights over forests have remained unimplemented in various regions of India (Kumar *et al.*^2015^). In much the same way, Parajuli *et al.* (2015) demonstrate that in Nepal’s community, forestry benefits of a co-management program are greater in rich households and that the poor pay the highest indirect costs (Parajuli *et al.*^2015^). Agrawal and Gupta (2005) have similarly shown that the richer, more powerful rural residents with higher-caste status tend to participate and profit most from community forestry projects (Agrawal and Gupta 2005). To summarize, if left alone, and much like in Kunene, the rich are typically successful in establishing institutional regimes that work in their interests.

However, and as Moore has already shown, states set the agenda and support specific governance models through legislation. Furthermore, bureaucrats and contracted NGOs help in implementing legislation at the local level as Lund (2007a) explained. In this context, a significant number of studies reveal a liaison between the state and local elites (Blaikie 2006; Ribot 2009; Scott 1998). For example, Agrawal and Chhatre (2006) found that powerful external state officials hinder the adoption of governance rules best suited to local conditions and that their presence has a negative effect on forestry management while simultaneously protecting elite interests (Agrawal and Chhatre 2006). Much in contrast, we found the Namibian state to be supportive of the poor and their social, economic, and environmental needs. The engagement of state representatives differs significantly between communities and only where they stay engaged and support those with less wealth and bargaining power, the p.h.o.c. rule remains. While the Namibian state is valued for being comparably uncorrupt, it might also be that the supportive role of bureaucrats is related to the fact that with community management of water costs, there are no profits to be shared out. Hence, opportunities and incentives to support a particular program that benefits the wealthy are low.

## Conclusion

This article has shown that institutional development in Namibian communities can only be understood at the intersection of different processes involving a myriad of actors. Its findings have some political implications. Over recent decades, nation states have increasingly withdrawn from local resource management, and self-governance (CBNRM) has become a guiding principle of many development efforts—the idea being that turning ownership and responsibility over to local communities will empower them and help to eradicate poverty (Blaikie 2006; Ribot 1999; Ribot *et al.*^2006^). We find that left alone, the rich typically succeed in establishing institutional regimes much to their own economic advantage. Only if the state remains in an active role, it can ensure the interests of the poor.

In this paper, I have described rural water management at one point in time in one region of Namibia. As I have explained, wealth and age are correlated and the young who oppose a flat rate rule today are likely to be rich in a decade or two. It remains an interesting challenge for further work to see whether these preferences are constant across time or whether they change with new social and economic positions. From the observations made, and the dominance of the economic motive, I would hypothesize the latter to be the case.

While I was able to show how “new” institutions are negotiated at present, further research should show the consequences these changes have. As we have seen, often a rule prevails in which the poor subsidize the water consumption of the rich. This might widen the gap between rich and poor, as initial simulations suggest (Schnegg *et al.*^2016^). However, state involvement and a proportional rule could lead to negative consequences for the poor if the rich households increasingly feel that once everyone pays for his or her precise water usage, everyone should also care for his or her other concerns. Should the wealthier part of the population in Kunene withdraw from the moral obligations and the support they channel into exchange networks, the consequences would be severe for those who have much less. To follow up the consequences of particular institutional regimes implemented for water management in the communities of Kunene is one of the most interesting and important future research tasks for understanding change and its economic, moral, and social potentials.

## Footnotes

1 Big men are influential individuals who do not have formal authority but command respect through personal skills.

2 In this paper, I used the terms *institutions* and *institutional regime* interchangeably to denote a set of rules that concern a specific social field, here water management.

3 Data collected by Linke in Fransfontein during the dry season 2011.

4 Interview conducted by MS, 10.9.2006 (Fransfontein area).

5 Interview conducted by DM, 13.10.2011 (Okangwati area).

6 All community names are synonyms.

7 Interview conducted by MS, 20.3.2014 (Fransfontein area).

8 Interview conducted by KG, 26.10.2011 (Otwani area).

9 Interview conducted by MS, 25.3.2014 (Fransfontein area).

10 The analysis treats the six cases with an attenuated p.h.o.c.-rule where the richer people pay more but not exactly according to the numbers owned as cases of a p.h.o.c.-rule. If they were treated as flat rate or excluded from the analysis, the correlation would be even higher.
